# Effects of Deformation Parameters on Phase Transformation of B1500HS High-Strength Steel During the Non-Isothermal Deformation Process

**DOI:** 10.3390/ma18122843

**Published:** 2025-06-17

**Authors:** Muyu Li, Dan Yao, Bin Li, Suilu Yue, Zhiyong Chen, Erzhou Ren, Ningning Wang, Chong Yang

**Affiliations:** 1 School of Intelligent Vehicle Engineering, Luoyang Institute of Science and Technology, Luoyang 471000, China; limuyu1949@163.com (M.L.); yslxmuhg@163.com (S.Y.); 1854@163.com (Z.C.); 2 School of Intelligent Manufacturing, Luoyang Institute of Science and Technology, Luoyang 471000, China; wangning@lit.edu.cn (N.W.); yangchong@xcu.edu.com (C.Y.); 3 Henan International Joint Laboratory of Cutting Tools and Precision Machining, Luoyang Institute of Science and Technology, Luoyang 471000, China; jyrez@lit.edu.cn

**Keywords:** high-strength steel, phase transformation, martensite, non-isothermal deformation, color metallograph

## Abstract

To investigate the effects of deformation parameters on the phase transformation of B1500HS high-strength steel, non-isothermal deformation tests were conducted on a Thermomaster-Z thermal mechanical simulator under different conditions in this study. Qualitative and quantitative investigations were carried out by analyzing the dilatation curves, color metallograph, and hardness data of deformed specimens. The results indicate that deformation can promote the formation of non-martensite. Higher initial deformation temperature and lower strain are beneficial for obtaining more martensite in the deformed high-strength steel and leading to higher martensite transformation temperatures. Meanwhile, the variation of strain rate has relatively small effects on the content and transformation temperature of martensite, and the effects do not show a singular trend.

## 1. Introduction

With the increasingly serious environmental pollution and energy crisis, energy conservation and environmental protection have become the development trend of the automotive industry. Reducing the weight of automobiles can decrease fuel consumption and greenhouse gas emissions. Therefore, various automobile manufacturers consider lightweight vehicles as their development direction, and hot stamping technology is one of the effective methods to achieve this goal [1,2,3]. Currently, this technology has been applied in the production of automotive components, such as A-pillar, B-pillar, and roof crossbeams [4,5]. The materials used in the hot stamping process are high-strength steels, represented by B1500HS and 22MnB5. Due to the addition of boron, these materials have good hardenability and can significantly improve overall strength and hardness, thereby reducing vehicle weight while enhancing collision safety [6,7,8].

During the hot stamping process, the high-strength steel plates are first austenitized in heating furnace and then formed and quenched in the mold. In this process, the high-strength steel undergoes phase transformation, and its microstructure transforms from ferrite and pearlite to martensite, which can greatly improve the strength of the steel [9,10]. Hot stamping is a complex thermal-mechanical-transformation coupled field; high temperature deformation can alter the phase transformation process and ultimately affect the microstructure and mechanical properties of the forming part [11]. Therefore, studying the influence of deformation parameters on the phase transformation of high-strength steel is of significance for the theoretical research and application of hot stamping technology.

The occurrence of phase transformation can lead to a change in specific volume, which can be reflected by the dilatation-temperature curve. Based on this principle, researchers have explored the effects of isothermal [12] and non-isothermal [13,14] deformation on the phase transformation of high-strength steel by comprehensive analysis of the dilatation curve and microstructure of the deformed specimen. However, when multiple phases coexist in the material, this analysis method is difficult to use when seeking to determine the volume fraction of each phase in the deformed specimen, which is not conducive to in-depth research on this issue [15].

In response to this situation, researchers have introduced surface hardness mapping technology to analyze the microstructure of deformed specimens [16,17]. The foundation of this technology is that each phase in iron-carbon alloys has its own hardness level. By analyzing the established hardness map, quantitative and qualitative analysis of the microstructure can be achieved, thereby analyzing the impact of deformation on phase transformation [18]. However, during the hardness measurement process, multiple phases may exist simultaneously in the indentations produced by the indenter of Vickers hardness scales, which may lead to errors in the analysis results of the microstructure.

To conduct more accurate quantitative and qualitative analysis of microstructures, researchers have proposed color mirroring technology [19,20]. The principle of this method is to form an interference film on the metal surface through chemical methods; the interference effect of light causes different microstructures of the material to present unique colors. This technology provides a new approach for analyzing the impact of high temperature deformation on phase transformation.

Based on the above analysis, color metallographic technology was taken as the analysis method of the microstructure in this study. By comprehensive analysis of dilatation data, color metallograph, and hardness, the effects of non-isothermal deformation parameters on phase transformation of B1500HS boron steel were explored. The above research can provide experimental evidence for constructing theoretical foundation for the non-isothermal hot stamping process.

## 2. Materials and Methods

In this study, B1500HS high-strength steel, produced by Baowu Iron and Steel Group, was selected, which has excellent formability and mechanical properties, and is widely used in the hot stamping process. The original microstructure of B1500HS contained ferrite and pearlite, and the chemical composition is given in Table 1 [21].

### 2.1. Non-Isothermal Deformation Test

The non-isothermal deformation tests were conducted on the Thermecmastor-Z thermal mechanical simulator (Fuji Denko Seiki Co., Ltd., Tokyo, Japan), which is equipped with a deformation unit designed for conducting simple uniaxial compression tests. This device can also heat the specimen through an induction heating system, measure the dilatation of the specimen through an LED optical automatic tracking system, and control the cooling process by adjusting the spraying speed of the protective gas. The cylindrical specimens used in tests were obtained through the wire cutting, with a length of 12 mm and a diameter of 8 mm. During the test, a K-type thermal couple was welded to the middle of the specimen to measure the temperature. In addition, the experiments were conducted under an inert gas protection environment to avoid oxidation.

**Figure 1:**
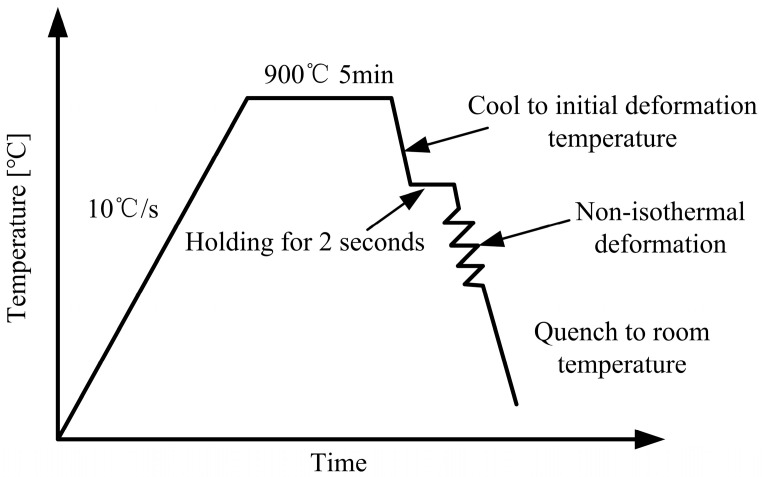
Schematic diagram of non-isothermal deformation test.

The schematic diagram of non-isothermal deformation test is shown in Figure 1. The specimens were heated to 900 °C with a heating rate of 10 °C/s and held for 5 min to ensure that they were completely austenitized. Subsequently, the specimens were cooled to the initial deformation temperature at a constant speed. To ensure the precise control of the deformation temperature, the specimen must be held isothermally at the initial deformation temperature for 2 s and then the compression started under different conditions. During and after the compression, the specimens were quenched to room temperature at a constant cooling rate, and the dilatation curves of deformed specimens were also measured. To analyze the effects of non-isothermal deformation parameters such as initial deformation temperature, strain, and strain rate on the phase transformation, three groups of tests were conducted. Detailed processing parameters are summarized in Table 2.

### 2.2. Microstructure

After the non-isothermal deformation tests, the microstructure of the deformed specimens were observed and analyzed to determine the influence of different deformation parameters on the phase transformation. Color metallographic technology was used to distinguish different phases in the deformed specimens. Metallographic specimens were cut from the middle of the deformed specimens by wire-electrode cutting and then ground and polished. Due to the high requirement of surface quality for color metallograph corrosion, the specimens need to be pre-corroded in a 4% nitric acid alcohol solution and then polished again to remove corrosion marks and obtain metallographic specimens with high surface quality. Afterwards, the Lepera reagent was prepared by mixing 10 g/L aqueous solution of sodium metabisulfite with 40 g/L ethanol solution of picric acid with a proportion of 1 to 1 [18], and the specimens were corroded in Lepera reagent for 20–40 s. When the color of the specimen changed, it was immediately removed and rinsed with alcohol. The colors of ferrite, bainite, and martensite in the corroded specimen are blue, brownish black, and white, respectively [19]. The microstructure of the corroded specimens was observed using an optical microscope (Sunny Instruments Co., Ltd., Ningbo, China), and we selected eight different locations to take metallographic images. Then, image analysis software Image Pro Plus 6.0 was used to quantitatively analyze the fraction of different phases in the specimen and the average of multiple measurement results were taken as the final data.

### 2.3. Hardness

The influence of non-isothermal deformation on the phase transformation can also be reflected in the mechanical properties of the material. The hardness tests were performed on the deformed specimens using a micro-hardness tester (Shanghai Haowei Photoelectric Technology Co., Ltd., Shanghai, China), with a load of 200 g and a holding time is 15 s. To reduce the measurement error, the hardness was measured at 10 different positions on the specimen and the average value was taken as the final result.

## 3. Results and Discussion

### 3.1. Effect of Strain

**Figure 2:**
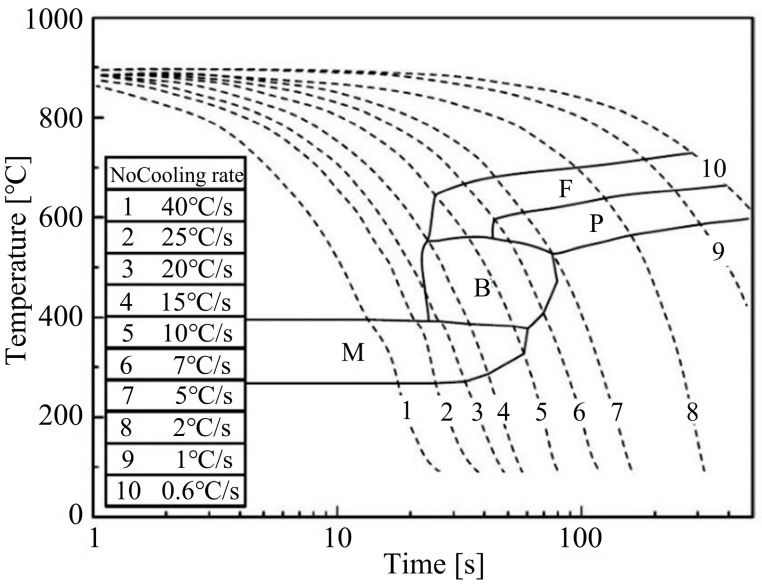
Continuous cooling transformation diagram of the B1500HS high-strength steel [14].

Figure 2 shows the continuous cooling transformation diagram of B1500HS high-strength steel. It can be concluded that when the cooling rate is higher than 25 °C/s, fully martensite can be obtained in the non-deformed steel.

**Figure 3:**
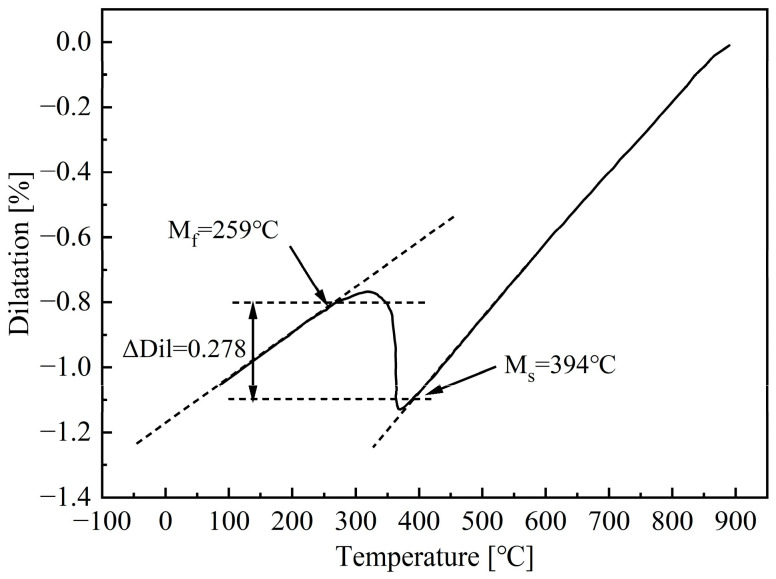
Dilatation-temperature curve of non-deformed B1500HS at a cooling rate of 30 °C/s.

Figure 3 shows the dilatation curve of the non-deformed specimen, with a cooling rate of 30 °C/s. From the figure, it can be seen that the dilatation curve decreases linearly with the decrease of temperature before reaching the martensite start temperature (M_s_), which indicates that no non-martensite is formed during the cooling process. The M_s_ and martensite finish temperatures (M_f_) of non-deformed B1500HS high-strength steel can be obtained by analyzing the dilatation curve with the tangent method, which are 394 °C and 259 °C, respectively. Since the occurrence of martensite transformation can cause fluctuations in the dilatation curve, according to References [12,16], the difference between the dilatation corresponding to M_s_ and M_f_ is an indicator to evaluate the degree of martensite transformation. From the figure, it can be determined that the change in dilatation caused by complete martensite transformation is ΔDil = 0.278%.

**Figure 4:**
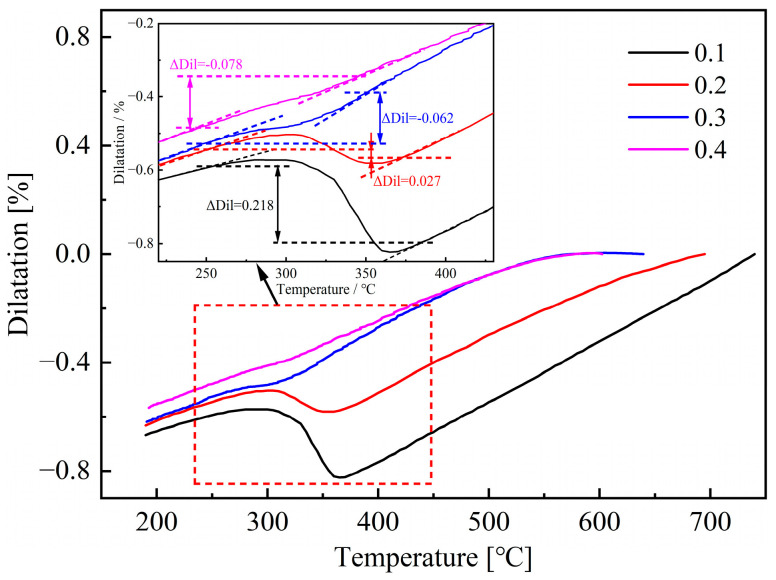
Dilatation curves after deformation at various strain and corresponding values of ΔDil.

The dilatation curves, after applying different amounts of strain under the initial deformation temperature of 800 °C and a strain rate of 0.1 s^−1^, are shown in Figure 4. It can be concluded that when the strain is 0.1, the shape of the measured dilatation curve is similar to that in Figure 3, indicating that there is basically no occurrence of non-martensite transformation under this deformation condition. As the strain increases from 0.2 to 0.4, significant nonlinear change can be observed in the dilatation curves when the temperature is higher than M_s_, which is caused by the formation of non-martensite. Based on the continuous cooling transformation diagram shown in Figure 2, it is preliminarily determined that the change in the dilatation curve is caused by the formation of ferrite and bainite.

The values of ΔDil measured from dilatation curves are also shown in Figure 4. It can be found that the measurement results decrease with the increase of applied strain, which indicates that the high temperature deformation can promote the formation of non-martensite, and this effect becomes more pronounced with the increase of strain.

**Figure 5:**
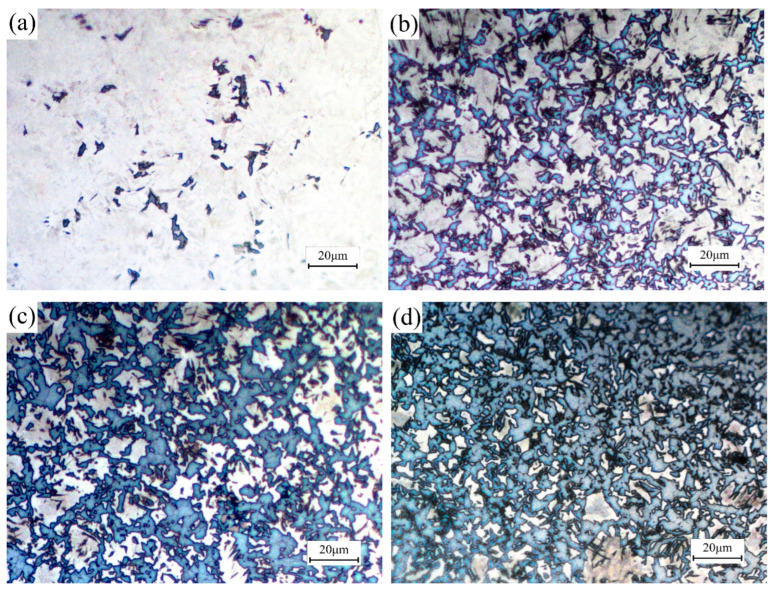
Microstructure of specimens deformed under different strains (**a**) 0.1; (**b**) 0.2; (**c**) 0.3; (**d**) 0.4.

To evaluate the effect of strain on the phase transformation from a microscopic perspective, Lepera reagent was used to corrode specimens obtained under different strains, and the metallographic images are shown in Figure 5. It can be observed that all the specimens contain non-martensite, and this phenomenon becomes more evident with the increase of strain. When the strain is 0.1, a small amount of blue ferrite exists in the microstructure of the specimen. When the strain is within the range of 0.2–0.4, the content of ferrite increases significantly with the increase of strain, and a small amount of brownish black bainite can also be observed.

**Figure 6:**
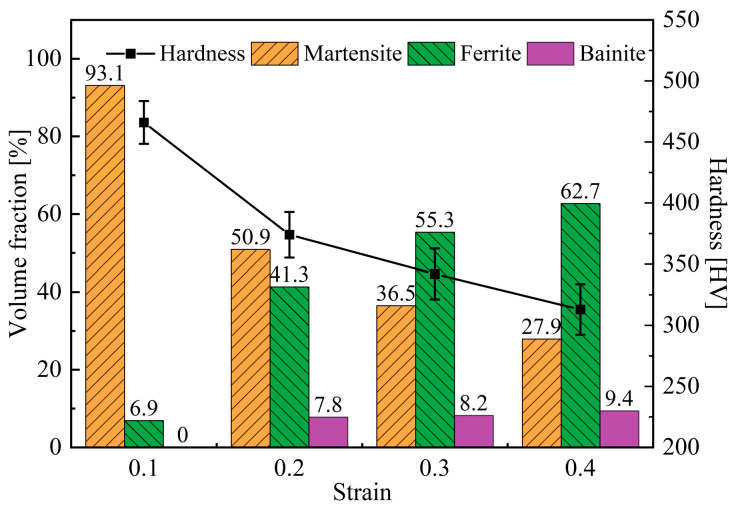
Microstructure composition and hardness of specimens after deformation under different strains.

By taking metallographic images at different positions and calculating the percentage of different colors with the image analysis software Image Pro Plus 6.0, quantitative analysis of different phases can be achieved; the analysis results are shown in Figure 6. From the figure, it can be seen that with the increase of strain, the martensite content decreases. On the contrary, the volume fraction of ferrite increases significantly. It can also be observed that as the strain increases from 0.1 to 0.2, bainite appears in the deformed specimen. After that, with the increase of strain, the volume fraction of bainite does not show a significant change. The difference in microstructure composition can be reflected in the hardness of the material. The measurement results shown in Figure 6 indicate that the increase of strain results in a reduction in hardness, which is mainly caused by the formation of ferrite.

Through the analysis of the above experimental results, it can be concluded that deformation has a promoting effect on the non-martensite transformation of B1500HS high-strength steel, which is due to the introduction of the stored deformation energy changing the original energy balance. The stored deformation energy mainly consists of grain boundary energy change and dislocation energy change. The non-isothermal deformation of austenite can increase the grain boundary area and enhance the disorder of the grain boundary structure, which results in higher grain boundary energy. In addition, the deformation can also lead to higher dislocation density, which can enhance the dislocation energy.

Existing research has shown that unless the strain reaches a relatively large magnitude, the grain boundary energy does not exhibit a significant increase [22]. Therefore, for hot stamping processes, the deformation-induced increase in grain boundary energy is negligible. When analyzing the impact of deformation on stored deformation energy, grain boundary energy can be ignored. The austenite dislocation energy per unit mole $\Delta G_{\mathrm{dis}}$ can be calculated by:

(1) $$\Delta G_{\mathrm{dis}}=\mu \rho b^{2}V_{\gamma },$$

where μ is the shear elastic modulus, ρ is the density of dislocation, *b* is the Burgers vector, and $V_{\gamma }$ is the molar volume of austenite.

The variation of dislocation density can be directly reflected in the flow stress. Under the condition of high temperature plastic deformation, the relationship between the flow stress and dislocation density can be expressed as [23]:

(2) $$\sigma =M\alpha \mu b\sqrt{\rho },$$

where *M* is Taylor factor, *α* is the material constant, and σ is the flow stress.

Without the consideration of grain boundary energy, the stored deformation energy $\Delta G_{D}$ can be approximately equal to $\Delta G_{\mathrm{dis}}$. By introducing Formula (2) into Formula (1), the relationship between the dislocation energy and flow stress can be obtained and represented as Formula (3). It can be seen from the formula that there is a positive correlation between the stored deformation energy and flow stress.

(3) $$\Delta G_{D}\approx \Delta G_{\mathrm{dis}}=\frac{\sigma^{2}V_{\gamma }}{M^{2}\alpha^{2}\mu^{2}},$$

Formula (4) shows the method for calculating the critical nucleation energy of ferrite, taking into account the stored deformation energy [22].

(4) $$\Delta G*=\frac{8\pi \sigma_{\gamma /\alpha }^{3}}{3{\left(\Delta G_{\mathrm{chem}}+\Delta G_{D}\right)}^{2}},$$

where $\sigma_{\gamma /\alpha }$ is the interfacial energy, $\Delta G_{\mathrm{chem}}$ is the chemical driving force, and ΔG* is the critical nucleation energy.

It is shown from Formula (4) that the introduction of stored deformation energy can reduce the critical nucleation energy required for ferrite transformation. According to the theory of solid-state phase transformation, the nucleation rate of ferrite can be calculated with the following formula [22]:

(5) $$I=K_{v}\exp\left(-\frac{\Delta G*}{kT}\right)\exp\left(-\frac{Q_{D}}{kT}\right),$$

where *I* is nucleation rate, $K_{v}$ is proportionality constant, $Q_{D}$ is the diffusion activation energy of Fe atoms in austenite, and k is the Boltzmann constant.

**Figure 7:**
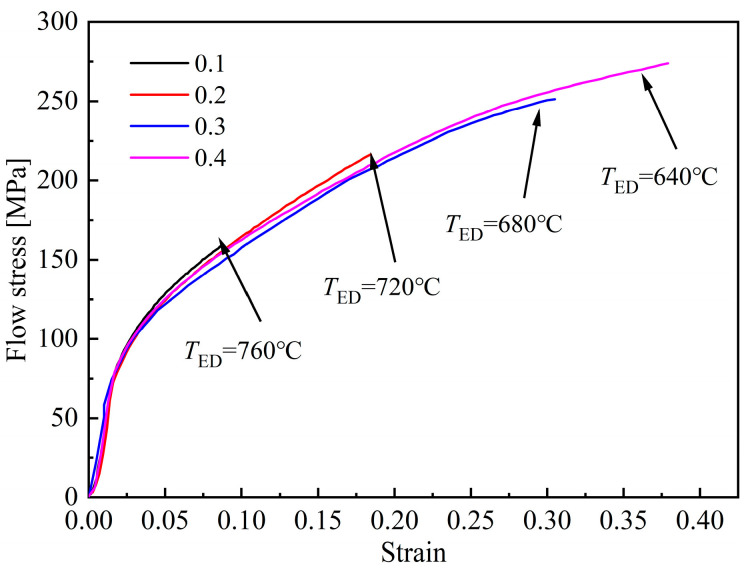
Flow stress-strain curves of specimens deformed at different strains.

By combining Formulas (4) and (5), it can be concluded that the increase of stored deformation energy can reduce the critical nucleation energy, thereby improving the nucleation rate and promoting the occurrence of ferrite transformation. The flow stress-strain curves measured during the deformation process are plotted in Figure 7; *T*_ED_ represents the ending temperature of deformation. With the increase of strain, the deformation finishes at lower *T*_ED_, and the flow stress corresponding to the end of deformation increases significantly. As a result, more stored deformation energy is generated, leading to a more obvious promoting effect on ferrite transformation, which is consistent with the quantitative analysis results shown in Figure 6.

Compared to ferrite, the variation of strain has no significant regular influence on the bainite transformation. This phenomenon is due to the fact that high temperature deformation has both promoting and inhibiting effects on bainite transformation. On the one hand, high temperature deformation can promote the nucleation of bainite and is thereby conducive to the formation of bainite. In addition, the external stress can reduce the interface energy and promote the segregation of carbon atoms at grain boundaries or crystal defect positions, thereby increasing nucleation rate and shortening the incubation period required for bainite transformation [24]. On the other hand, the dislocations formed during the deformation process can hinder the directional growth of bainite, and this effect becomes more pronounced with the increase of dislocation density [13].

**Figure 8:**
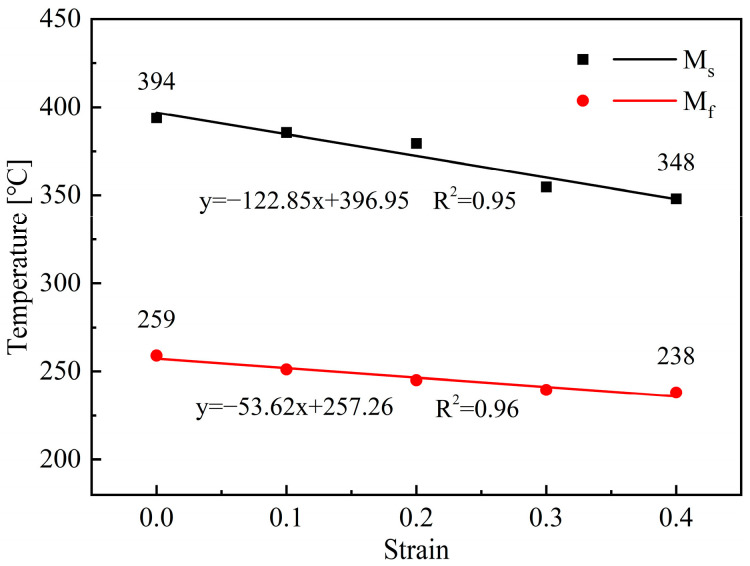
Effect of strain on the M_s_ and M_f_ temperatures.

By analyzing the dilatation curves shown in Figure 4 with the tangent method, the M_s_ and M_f_ temperatures under different strain conditions can be obtained, and the results are shown in Figure 8. It can be concluded that the increase of applied strain can lower the M_s_ and M_f_ temperatures, and the downward trend of M_s_ is more pronounced. During the deformation process, the generated crystal defects can hinder the movement of atoms, thereby improving the mechanical stability of austenite and hindering the martensite transformation, and this phenomenon becomes more pronounced with the increase of strain. Therefore, larger undercooling is needed to enhance the driving force of martensite transformation, resulting in a decrease in M_s_ and M_f_ temperature. Furthermore, according to the quantitative analysis results shown in Figure 6, the increase of strain can promote the transformation of ferrite, resulting in carbon enrichment in the remaining austenite and ultimately reducing the M_s_ and M_f_ temperatures.

### 3.2. Effect of Initial Deformation Temperature

**Figure 9:**
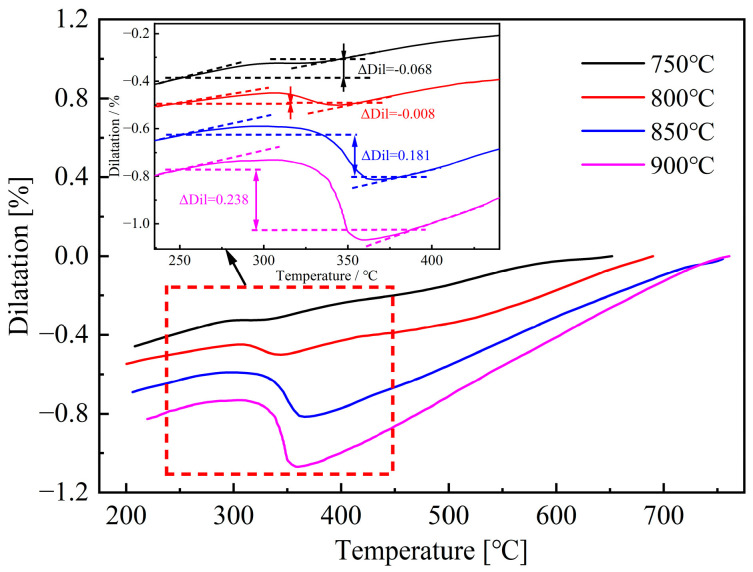
Dilatation curves after deformation at various initial deformation temperatures and corresponding values of ΔDil.

Dilatation curves obtained under different initial deformation temperatures are shown in Figure 9, and the changes in dilatation caused by martensite transformation are listed in the figure. When the initial deformation temperature is 750 °C and 800 °C, the fluctuation caused by the formation of bainite and ferrite can be clearly observed in the dilatation curves. On the contrary, this phenomenon weakens when the initial deformation temperature reaches 900 °C and 850 °C.

By analyzing the data of ΔDil shown in Figure 9, it can be observed that the values are all less than 0.278%, which indicates the existence of non-martensite in the deformed specimens. Furthermore, it can also be concluded that there is a positive correlation between the initial deformation temperature and the value of ΔDil. Higher initial deformation temperature is conducive to obtaining more martensite.

**Figure 10:**
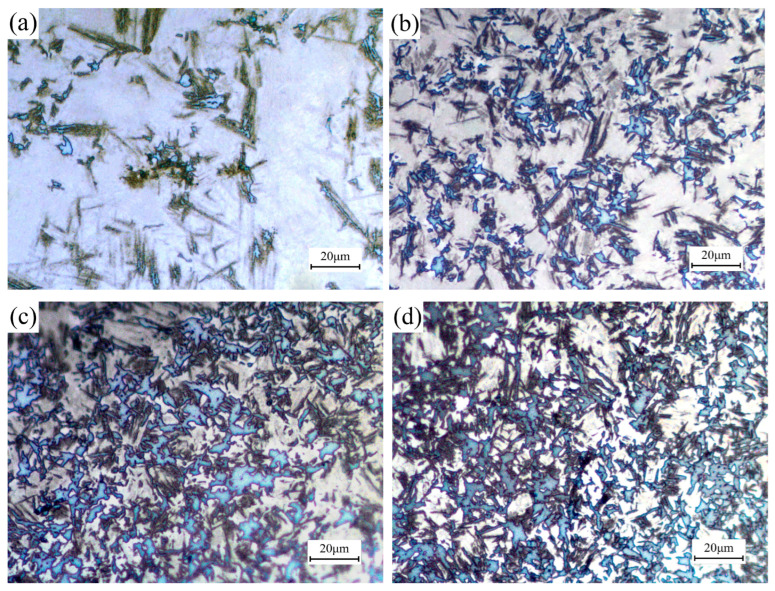
Microstructures of specimens deformed under different initial deformation temperatures: (**a**) 900 °C; (**b**) 850 °C; (**c**) 800 °C; (**d**) 750 °C.

Figure 10 shows the metallographic images of B1500HS high-strength steel after the non-isothermal deformation under different initial deformation temperatures. From observation, it can be inferred that the microstructure of all deformed specimens is composed of ferrite, bainite, and martensite. The volume fraction of martensite significantly decreases with the reduction of initial deformation temperature, which is consistent with the analysis results obtained from dilatation curves.

**Figure 11:**
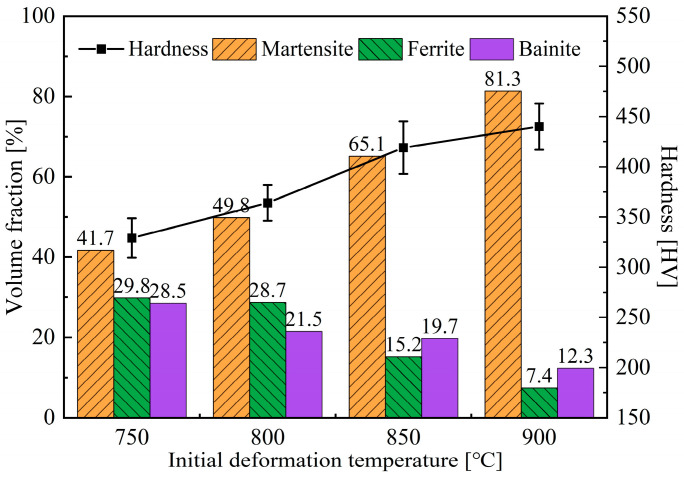
Microstructure composition and hardness of specimens after deformation under different initial deformation temperatures.

Quantitative analyses of microstructure and hardness measurement were conducted on specimens obtained under different initial deformation temperatures, and the results are shown in Figure 11. It can be concluded that the higher initial deformation temperature can suppress the formation of ferrite and bainite, significantly increase the volume fraction of martensite, and thus cause an increase in hardness.

**Figure 12:**
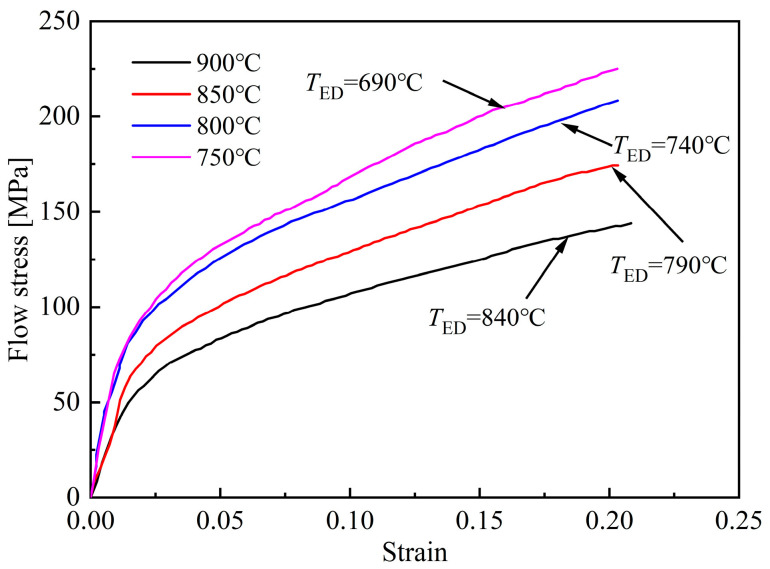
Flow stress-strain curves of specimens deformed at different initial deformation temperatures.

Figure 12 shows the flow stress-strain curves obtained under different initial deformation temperatures. With the increase of the initial deformation temperature, the deformation occurred in a higher temperature range, and the softening effect, caused by dynamic recrystallization (DRX) and dynamic recovery (DRV), becomes more obvious, thus reducing the strength of austenite [21]. By increasing the initial deformation temperature, the stored deformation energy decreases, thereby weakening the promoting effect on ferrite and bainite transformation and resulting in higher martensite content.

**Figure 13:**
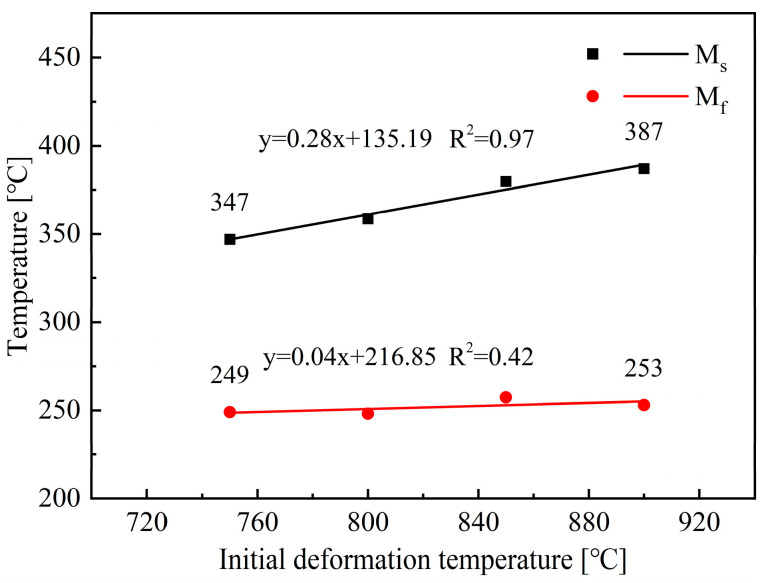
Effect of initial deformation temperature on the M_s_ and M_f_ temperatures.

By analyzing the dilatation curves shown in Figure 9, M_s_ and M_f_ temperatures can be obtained, as shown in Figure 13. The result shows that increasing the initial deformation temperature from 750 to 900 °C can lead to a higher M_s_ temperature. In contrast, the variation of initial deformation temperature has no obvious impact on M_f_ temperature. The increase in initial deformation temperature suppresses the formation of non-martensite, the carbon content of the remaining austenite decreases, and therefore leads to higher M_s_ temperatures. In addition, deformation at higher temperatures can promote the occurrence of DRX and DRV, thereby reducing the shear strength of austenite, which is also beneficial for increasing the martensite transformation temperature [10,25].

### 3.3. Effect of Strain Rate

**Figure 14:**
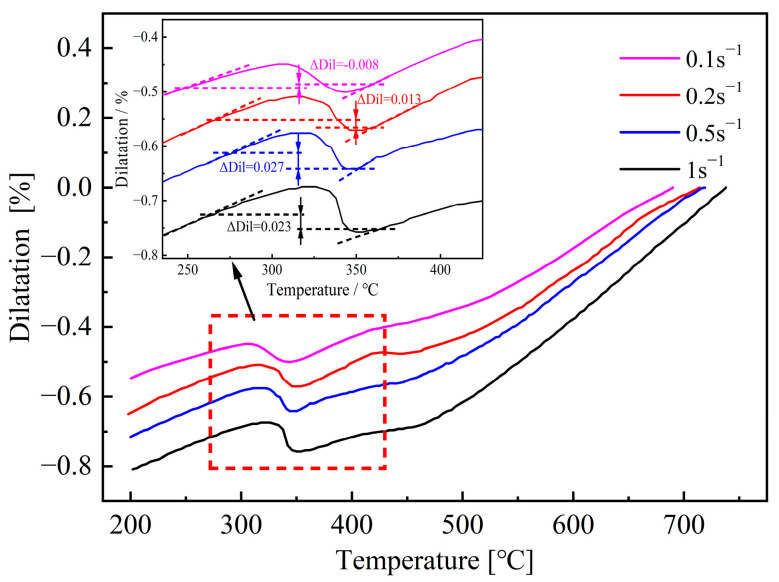
Dilatation curves after deformation at various strain rates and corresponding values of ΔDil.

Figure 14 shows the dilatation curves measured under the strain rates of 0.1 s^−1^, 0.2 s^−1^, 0.5 s^−1^, and 1 s^−1^. Although the strain rates are different, the dilatation curves have similar shapes, and the change in dilatation caused by the formation of bainite and martensite can be clearly observed. In addition, within the range of 0.1 s^−1^ and 0.5 s^−1^, the increase of strain rate can slightly increase the value of ΔDil, which indicates more martensite being formed in the deformed specimens. However, when the strain rate increases to 1 s^−1^, a lower ΔDil can be obtained, which implies that the martensite transformation is suppressed.

**Figure 15:**
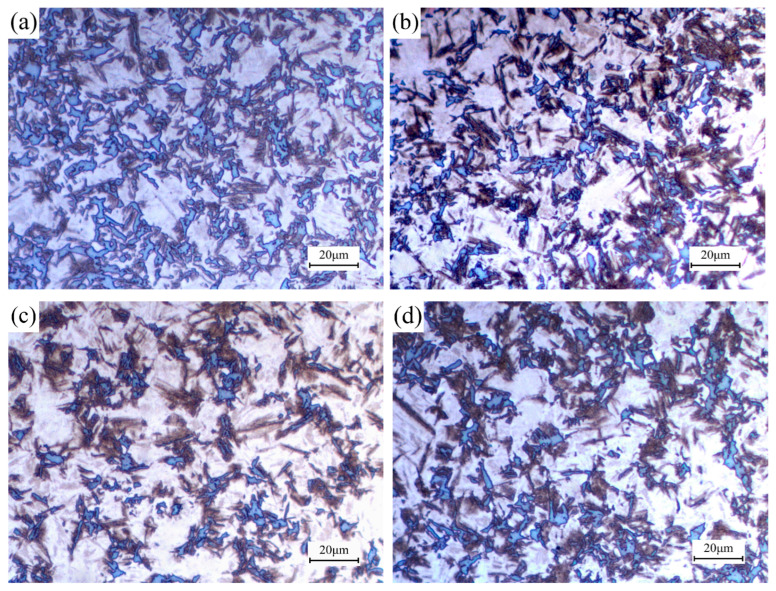
Microstructures of specimens deformed under different strain rates: (**a**) 0.1 s^−1^; (**b**) 0.2 s^−1^; (**c**) 0.5 s^−1^; (**d**) 1.0 s^−1^.

**Figure 16:**
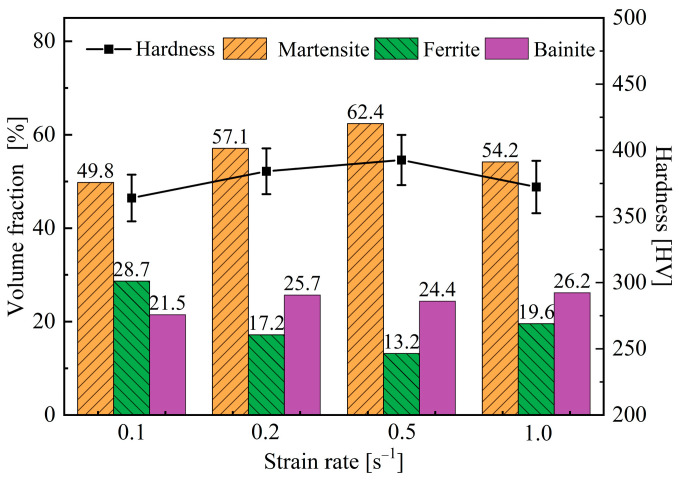
Microstructure composition and hardness of specimens after deformation under different strain rates.

The metallographic images of B1500HS high-strength steel after non-isothermal treatment under different strain rates are shown in Figure 15. It can be observed that the microstructure of the deformed specimens is composed of ferrite, bainite, and martensite. Quantitative analyses have been conducted and the results are shown in Figure 16. Within the range of 0.1~0.5 s^−1^, the increase of strain rate is beneficial for improving the volume fraction of martensite, while the volume fraction of ferrite decreases with the increase of strain rate; this phenomenon is most pronounced when the strain rate increases from 0.1 s^−1^ to 0.2 s^−1^. Afterwards, the strain rate increases from 0.5 s^−1^ to 1.0 s^−1^, the volume fraction of martensite decreases, and more ferrite is formed. In addition, it can be seen from the figure that the volume fraction of bainite changes with the increase of strain rate, but the magnitude is not significant and has no obvious regularity. It can also be seen from the figure that the hardness and martensite content changes in the same trend, with the increase of strain rate, hardness first being increased and then decreased.

**Figure 17:**
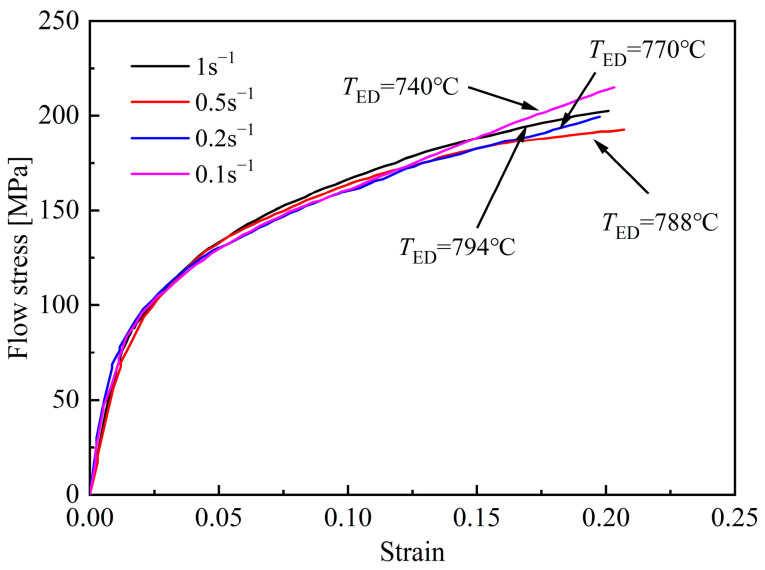
Flow stress-strain curves of specimens deformed at different stain rates.

Based on the analysis above, it can be concluded that the effect of strain rate on phase transformation of B1500HS is not singular. The flow stress-strain curves, measured during the deformation process, can be used to explain the reason for this phenomenon, and the curves are plotted in Figure 17. For non-isothermal deformation, the strain rate has two opposite effects on the strength of austenite: (1) The increase of strain rate can improve the *T*_ED_, which is beneficial to reduce the deformation resistance; (2) Higher strain rate leads to the improvement of dislocation density, which is detrimental to the softening effect caused by DRX and DRV, resulting in higher deformation resistance.

Within the range of 0.1–0.5 s^−1^, the flow stress decreases with the increase of strain rate, which indicates that the increase of *T*_ED_ has more significant impact on the deformation resistance. The reduction of flow stress weakens the promoting effect on ferrite transformation, which is consistent with the quantitative analysis results shown in Figure 16. When the strain rate increases to 1 s^−1^, the main factor affecting the flow stress is the enhancement of the strain rate. As a result, the flow stress increases and more ferrite is induced. In addition, compared to the flow stress-strain curves shown in Figure 7 and Figure 12, it can be seen that the variation in flow stress caused by strain rate is smaller than that caused by initial deformation temperature and strain, which means that the promoting effect of strain rate on ferrite transformation is smaller than that of strain and initial deformation temperature. This conclusion can be verified by the analysis results of microstructure.

**Figure 18:**
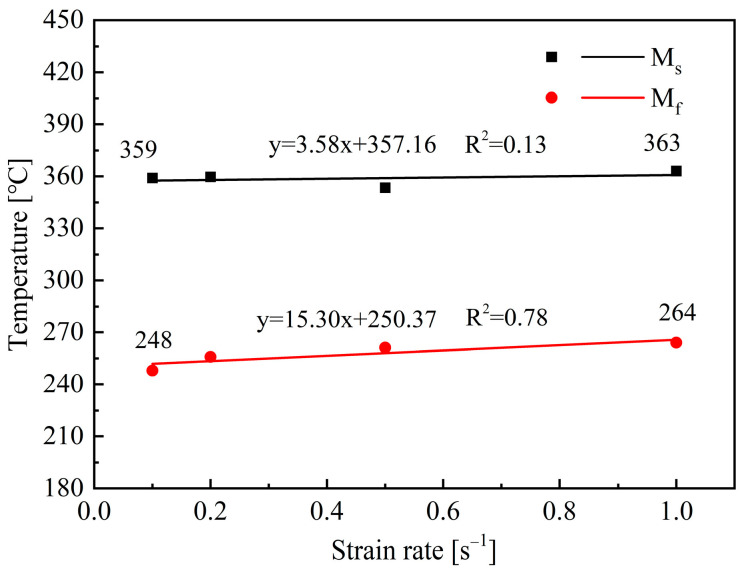
Effect of strain rate on the M_s_ and M_f_ temperatures.

Figure 18 shows the M_s_ and M_f_ temperatures under different strain rates. It indicates that increasing the strain rate from 0.1 to 1.0 s^−1^ can slightly increase the M_f_ from 248 °C to 264 °C. Meanwhile, the influence of strain rate on M_s_ can be negligible. Compared to the other two factors, the change in strain rate has a relatively small impact on the phase transformation, as well as the flow stress during deformation. Therefore, the stability of the remaining austenite is less affected, and the variation of martensite transformation temperature is relatively small.

## 4. Conclusions

In the current article, the effects of strain, initial deformation temperature, and strain rate on phase transformation of B1500HS high-strength steel under non-isothermal deformation conditions were investigated, and the following conclusions can be made.

(1) The stored deformation energy introduced by non-isothermal deformation can promote the transformation of ferrite. Through the quantitative analysis of microstructure, as the strain increases and the initial deformation temperature decreases, more ferrite is present in the deformed specimen, which leads to the decrease in hardness.
(2) The effect of strain rate on ferrite phase transformation is not a single trend. When the strain rate increases from 0.1 to 0.5 s^−1^, the enhancement of *T*_ED_ leads to the decrease in deformation resistance, which reduces the promoting effect on ferrite transformation, resulting in the decrease of ferrite content. Afterwards, the increase in strain rate plays a dominant role in the variation of deformation resistance, and the increase of strain rate can promote the formation of ferrite.
(3) The variation of deformation parameters can also cause the fluctuation of bainite content. The increase of strain and the decrease of initial deformation temperature can also enhance the content of bainite in the deformed specimens, but the effect is weaker than that on ferrite. The reason is that the non-isothermal deformation can not only promote the nucleation process of bainite but also have an inhibitory effect on the growth of it. Under the combined influence of the two opposite effects, the volume fraction of bainite does not change significantly.
(4) Non-isothermal deformation can improve the stability of austenite, thus reducing the M_s_ temperature of B1500HS high-strength steel. With the increase of strain and decrease of initial deformation temperature, lower M_s_ temperature can be obtained. Meanwhile, the variation of strain rate has no significant impact on M_s_ temperature. Compared to the M_s_ temperature, the parameters mentioned above have less impact on the M_f_.

The results of this article can provide a basis for determining the hot stamping process of B1500HS high-strength steel. However, in this article, authors did not analyze the influence of cooling rate during the deformation process on subsequent phase transformation, which is also one of the main factors affecting the quality of hot stamping products. The above content will be further studied in the future.

## Figures and Tables

**Table 1:** Chemical composition of B1500HS [21] (wt%).

C	Si	Mn	Cr	Mo	B	Ti	V	S	P
0.23	0.25	1.35	0.19	0.04	0.003	0.03	0.004	0.006	0.015

**Table 2:** Processing parameters of non-isothermal deformations applied to B1500HS steel.

No.	Amount of Strain	Strain Rate [s^−1^]	Cooling Rate [°C/s]	Initial Deformation Temperature [°C]
1	0.1, 0.2, 0.3, 0.4	0.1	40	800
2	0.2	0.1	30	750, 800, 850, 900
3	0.2	0.1, 0.2, 0.5, 1.0	30	800

## Data Availability

The original contributions presented in this study are included in the article. Further inquiries can be directed to the corresponding authors.

## Nomenclature

M_s_	martensite start temperature
M_f_	martensite finish temperature
ΔDil	change in dilatation caused by martensite transformation
$\Delta G_{\mathrm{dis}}$	austenite dislocation energy per unit mole
μ	shear elastic modulus
ρ	density of dislocation
b	Burgers vector
$V_{\gamma }$	molar volume of austenite
*M*	Taylor factor
*α*	material constant
σ	flow stress
$\sigma_{\gamma /\alpha }$	interfacial energy
$\Delta G_{\mathrm{chem}}$	chemical driving force
ΔG*	critical nucleation energy
I	nucleation rate
$K_{v}$	proportionality constant
$Q_{D}$	diffusion activation energy of Fe atoms in austenite
k	Boltzmann constant
*T*_ED_	ending temperature of deformation
